# Mitochondria-derived vesicles and their potential roles in kidney stone disease

**DOI:** 10.1186/s12967-023-04133-3

**Published:** 2023-05-02

**Authors:** Sakdithep Chaiyarit, Visith Thongboonkerd

**Affiliations:** Medical Proteomics Unit, Research Department, Faculty of Medicine Siriraj Hospital, Mahidol University, 6th Floor, SiMR Building, 2 Wanglang Road, Bangkoknoi, Bangkok, 10700 Thailand

**Keywords:** EVs, Extracellular vesicles, MDVs, Mitovesicles, Nephrolithiasis, Oxidative stress, Urolithiasis

## Abstract

Recent evidence has shown significant roles of mitochondria-derived vesicles (MDVs) in mitochondrial quality control (MQC) system. Under mild stress condition, MDVs are formed to carry the malfunctioned mitochondrial components, such as mitochondrial DNA (mtDNA), peptides, proteins and lipids, to be eliminated to restore normal mitochondrial structure and functions. Under severe oxidative stress condition, mitochondrial dynamics (fission/fusion) and mitophagy are predominantly activated to rescue mitochondrial structure and functions. Additionally, MDVs generation can be also triggered as the major MQC machinery to cope with unhealthy mitochondria when mitophagy is unsuccessful for eliminating the damaged mitochondria or mitochondrial fission/fusion fail to recover the mitochondrial structure and functions. This review summarizes the current knowledge on MDVs and discuss their roles in physiologic and pathophysiologic conditions. In addition, the potential clinical relevance of MDVs in therapeutics and diagnostics of kidney stone disease (KSD) are emphasized.

## Introduction

Vesicular transport is a regulatory mechanism in all living cells. The cell-derived vesicles are originated from various cellular organelles, including mitochondria. Several lines of evidence have demonstrated essential roles of mitochondria-derived vesicles (MDVs) in mitochondrial quality control (MQC) system [1–4]. This control system is crucial for mitochondrial homeostasis and cell survival regulation [5, 6]. As such, MDVs formation in the MQC system is recognized as the first-line and vital regulatory mechanism in both physiologic and pathologic conditions [2, 7]. Novel findings of MDVs generation during the past decade have amplified our understanding of non-mitophagy pathway for mitochondrial preservation and cell survival. Many lines of MDVs research have shown greater MDVs level in mild stress conditions than mitophagy, which is the canonical machinery for removing the damaged mitochondria [1, 8–11]. Recently, selective cargos of mitochondrial oxidized molecules, such as mitochondrial DNA (mtDNA), peptides, proteins and lipids, to be degraded by lysosomes have been shown [3, 12, 13]. Moreover, immune regulation by MDVs has been emphasized in several reports of inflammation-associated diseases [3, 12, 14]. As such, MDVs have gained a wide interest in many mitochondria-associated disorders/diseases, such as cancers [15, 16], aging [17–19], cardiovascular diseases [20, 21], and neurodegenerative disorders [22–24].

It is well known that kidney stone disease (KSD) is associated with oxidative stress and mitochondrial abnormalities in renal tissue [25–29]. Cellular mechanisms of mitochondrial dysfunction associated with kidney stone formation have been proposed [25]. For example, renal tubular inflammation and peroxidation of lipids and proteins in cell membranes induced by mitochondrial abnormalities can increase crystal deposition in the kidney [25]. Components of dead cells and fragmented organelles, including mitochondria, also serve as the sources for stone nidus (core component) formation [25]. Additionally, the damaged mitochondria can promote renal interstitial inflammation that further enhances development and formation of the Randall’s plaque, which is one of the common pathologies serving as the nidus for calcium oxalate (CaOx) kidney stone [25]. Therefore, preserving mitochondrial functions has been proposed as one of the preventive strategies against KSD [25].

In addition to the whole mitochondria and their fragments, several lines of recent evidence have implicated the involvement of intracellular and extracellular MDVs in kidney stone formation. This review therefore summarizes the current knowledge on roles of MDVs, particularly in KSD.

## Overview of MDVs

The evolutionary origin of mitochondria is from archaebacteria that ordinarily transport vesicles in order to communicate with other living microorganisms, escape from host immune systems, and eliminate self-damaged materials [30, 31]. Thus, MDVs formation has been proposed as the ancient homeostatic process in living cells at mitochondrial level under physiologic and mild stress conditions [21, 32]. Although removal of the damaged mitochondria or mitochondrial contents by autophagy in the MQC system for cell homeostasis has been extensively studied [5, 6], several mechanisms of mitochondrial reinforcement and repair remain unclear. Hence, recent concepts of micromitophagy [33, 34], MDVs formation [1, 8, 10, 22], and mitophagy-independent machinery [35–37] have been emerged to explain mitochondrial stability [8], prevention of cell death [37] and tissue repair [38, 39].

The intracellular vesicles that contain mitochondrial components have been recognized as mitochondrial vesicles or MDVs [40]. They are the nanoscale vesicles (approximately 70–150 nm in diameter) surrounded by single or double membranes, i.e., outer mitochondrial membrane (OMM) and/or inner mitochondrial membrane (IMM) [7, 11, 22]. MDVs are also the specific cargos for mitochondrial nucleic acids (DNA and RNA) [3, 21, 41–45], proteins [3, 22, 46, 47], lipids [7, 32, 37], fragmented mitochondria [5, 48] and/or other mitochondrial components [49–51]. Previous studies have shown that MDVs play major roles in intracellular interactions of the parental mitochondria with lysosomes [44, 52], endosomes [7, 44], and peroxisomes [22, 53]. Additional reports have demonstrated intercellular roles of MDVs in removing malfunctioned part of mitochondria [3, 44, 54], transferring functional MDVs to communicate with the target cells that require more energy [55–57] and regulating immune response [58, 59].

MDVs are known as the key component of the first-line secure process in the MQC system, and their possible roles entirely differ from mitochondrial dynamics (fission/fusion) and mitophagy [1, 4, 5, 10]. Additionally, the number of MDVs is increased by mild stress or early stage of mitochondrial dysfunction [21]. Two main types of MDVs have been recognized in the MQC system, including steady-state MDVs [32, 60] and stress-induced MDVs [8, 39], both of which can be characterized by their specific markers. Translocase of outer mitochondrial membrane 20 (TOMM20), an OMM protein, is mostly found in steady-state MDVs (TOMM^+^-MDVs) [32], whereas pyruvate dehydrogenase (PDH) is predominantly found in oxidative stress-triggered MDVs (PDH^+^-MDVs) [61]. Unveiling the MDVs formation and their functional roles would make the image of mitochondria-related intracellular and intercellular communications much clearer.

## Biogenesis of MDVs

Previously, mitochondrial membrane blebbing and mitophagy-related machinery had been proposed as the possible mechanisms for MDVs formation [7]. However, later evidence has clearly shown that MDVs are independent of mitochondrial dynamics and mitophagy [5, 40]. One of the newly proposed mechanisms for MDVs biogenesis is via PINK1 (phosphatase and tensin homolog-induced kinase 1)/Parkin (an E3 ubiquitin protein ligase containing ubiquitin-like domain at N-terminus)-dependent, but DRP1 (dynamin related protein 1)-independent process [7, 52, 61, 62]. In mild stress condition or slight mitochondrial damage, mitochondrial membrane curvature is initiated followed by PINK1 accumulation [8, 10, 40]. Parkin is then recruited at OMM, and the MDVs are scissored and released by an unclear mechanism [7, 8, 10, 40, 52]. The involvement of DRP1 in MDVs generation has been excluded as MDVs can be formed even when DRP1 is knocked down [40].

By contrast, several investigations have shown that MDVs can be formed in PINK1-deficient cells [4, 7, 62, 63]. Recent proteome study has documented a new molecular model of MDVs biogenesis in resting stage that depends on the microtubule-associated motor proteins, MIRO1 and MIRO2 (MIRO1/2), and DRP1-dependent mechanism for cutting and releasing MDVs from parental mitochondria, whereas Parkin and PINK1 are not involved in this mediated pathway [32]. MDVs formation begins at steady-state by mitochondrial membrane protrusion after MIRO1/2 formation followed by recruitment of DRP1 by 49- and 51-kDa mitochondrial dynamics DRP1 receptor protein (MiD49 and MiD51, respectively) or mitochondrial fission factor (MFF) [32]. To complete MDVs construction, DRP1 then catalyzes the cutting of thin membrane tube to release MDVs that can be delivered to their specific targets. However, further elucidations for precise mechanism are needed as this group of the investigators have previously demonstrated that DRP1 silencing does not affect MDVs formation [21, 52, 61, 64] (in contrast to their own recent findings). They have described that the contradictory results were due to dissimilar gene knockout technique in each work. DRP1 was > 95% silencing in the prior study by simple molecular technique but was completely deleted by a more effective method, namely clustered regularly interspaced short palindromic repeats (CRISPR)/CRISPR-associated (Cas) system, in a recent work [32]. Thus, MDVs can be formed in an incomplete DRP1-knockdown condition. Nevertheless, they have also suggested that the steady-state MDVs formation does not require Parkin and PINK1, which may be needed for generation and regulation of MDVs formation during oxidative stress and inflammatory conditions [48, 65]. Moreover, the dynamicity of MDVs formation may be also affected by techniques of detection, isolation, and diverse states of diseases or study models. Therefore, future studies on MDVs should clearly provide sufficient details of methodology and conditioning used in each study for clarification. And more extensive investigations are required for further elucidations of the precise mechanism(s) of MDVs biogenesis.

## Classification and subtypes of MDVs

Most of the investigations on MDVs have been done inside the cells with their inter-organellar interactions [8, 38, 40]. However, MDVs are considerably diverse. Immuno-labelling together with high-resolution electron microscopy [4, 21, 57, 60, 66], proteomics and lipidomic profiling [22, 32, 46, 67] can enhance the study of MDVs. Currently, intracellular MDVs can be discriminated from other intracellular vesicles by using their specific markers, including OMM, IMM, mitochondrial matrix proteins and mtDNA [2, 7].

In addition to the intracellular MDVs, increasing evidence of extracellular MDVs has been documented. The secretion of extracellular MDVs has been suggested to be associated with endolysosomal and multivesicular body (MVB) formation, a mechanism that is similar to secretion of extracellular vesicles (EVs) [7, 11, 40, 46, 47, 68–73]. In general, EVs are classified based-on their diameter, biogenesis mechanism and specific protein markers. These EVs commonly include exosomes, microvesicles (MVs) and apoptotic bodies (ABs) [74, 75]. ABs are macrovesicles that are secreted from apoptotic cells during cell death by apoptotic mechanism [76]. The size of ABs extremely differs from that of MDVs. However, diameters of MVs and exosomes are approximately 100–1000 nm [77, 78] and 20–200 nm [79, 80], respectively, which overlap with that of MDVs (50–150 nm) [60]. As such, MVs can be discriminated from MDVs by their MVB-independent secretory mechanism [81, 82]. Nevertheless, exosomal secretion is MVB-dependent [83, 84] similar to that of MDVs [7, 40, 46, 68]. Thus, extracellular MDVs can be discriminated from exosomes by using corresponding specific markers. To discriminate the isolated MDVs from EVs by their differential size, high-resolution nanoparticle tracking analysis (NTA) is the method of choice [85–87]. Excluding MVs and exosomes with size overlapping that of MDVs would require high-resolution isolation and specific detection of mitochondrial components such as IMM, OMM, mitochondrial matrix proteins and mtDNA [7, 40, 46, 68, 88–90].

**Fig. 1:**
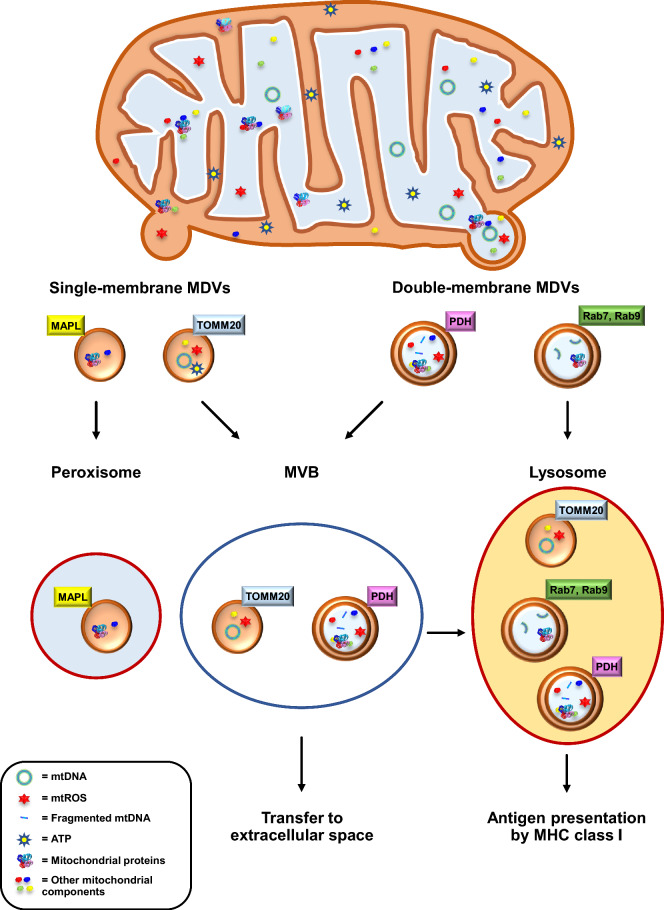
Classification and subtypes of MDVs. MDVs can be classified based-on their membranes and specific cargos. The single-membrane MDVs contain outer mitochondrial membrane (OMM) proteins, whereas double-membrane MDVs contain OMM and inner mitochondrial membrane (IMM) proteins as well as mitochondrial matrix proteins. Based on these different cargos, there are specific protein markers for subtype classification. Mitochondria-anchored protein ligase (MAPL) and translocase of outer mitochondrial membrane 20 (TOMM20) are the common markers for single-membrane MDVs. Peroxisome is the terminal of MALP^+^-MDVs, while TOMM^+^-MDVs are excreted by multivesicular body (MVB) process like exosomes. Pyruvate dehydrogenase (PDH) are the specific protein marker for double-membrane MDVs, which are excreted by the MVB process. Moreover, MDVs formation in the presence of Rab7 (a small GTPase that monitors vesicular transport to late endosomes and lysosomes) and Rab9 can mediate antigen presentation via MHC class I

Packaging of MDVs is a complex mechanism associated with their diverse functions and destinations. Therefore, MDVs subtypes may be classified based on their specific contents and targets (Fig. 1). For example, MDVs containing mitochondria-anchored protein ligase (MAPL) are transported to peroxisomes [64, 91]. Similarly, MDVs containing peroxisomal biogenesis factor 3 (Pex3) and peroxisomal biogenesis factor 14 (Pex14) play crucial roles in peroxisomal biogenesis [92, 93]. Although MDVs containing Pex3/Pex14 or MAPL share the same targets, each of them functions differently.

Additionally, MDVs can be classified based on the cellular status, including steady-state MDVs and stress-induced MDVs, which are the two distinct subtypes of MDVs widely investigated in several disease models [8, 32, 39, 60]. The steady-state MDVs are typically demonstrated as TOMM^+^/PDH^−^ MDVs, whereas TOMM^−^/PDH^+^ MDVs (stress-induced MDVs) are predominantly found during oxidative stress [32, 61]. The biogenesis of the steady-state MDVs is PINK1/Parkin-independent, in contrast to that of the stress-induced MDVs as discussed above [52, 61]. After biogenesis, both TOMM^+^/PDH^−^ and TOMM^−^/PDH^+^ MDVs carry the damaged mitochondrial components and transfer them to lysosomes for degradation to maintain mitochondrial structure and functions.

Another subtype of extracellular nanovesicles that correlate with MDVs has been recently isolated by high-resolution density gradient separation and termed as “mitovesicles” [68, 70]. Their size is approximately 6 nm and differs from other subtypes of MDVs or EVs. Mitovesicles are small double-membrane EVs that contain proteins involved in catabolic pathway, energy production and pro-fission process, but lack of proteins involved in biosynthesis, transport and pro-fusion process [19, 68]. Mechanisms of mitovesicles formation and release to extracellular space are not specified at this stage, but has been postulated to fuse with MVB before being secreted from the cells [68]. Moreover, mitovesicles serve as the functional vesicles based on the inside mitochondrial components [19, 68].

Although several subtypes of MDVs have been reported, their molecular machineries and biogenesis remain not well understood. Hence, specific cargos, functions, targets and subtypes of MDVs still require further elucidations for clarification.

## Roles of MDVs in physiology and pathophysiology

Under physiologic state with mild stress, MDVs serve as a part of the crucial process in the MQC system to preserve mitochondrial functions [2, 21, 91]. MDVs formation has been proposed as the first-line mitochondrial safety to remove damaged mitochondrial components prior to detrimental derangement of the entire mitochondria and cell death activation [1, 5, 8, 38, 40, 46]. In addition, the increase of MDVs is the finest compensatory mechanism of the MQC system, when mitophagy does not work to eliminate the impaired mitochondria [1, 37, 63]. Thereafter, biogenesis of mitochondrial proteins and lipids is activated to restore the mitochondrial functions [5, 7, 94]. MDVs are therefore considered as a novel potential therapeutic target for maintaining the MQC system and preventing mitochondrial dysfunction in normal and disease conditions. MDVs also get involved in communications between mitochondria and other intracellular organelles. They not only transport the damaged compartments to endolysosomes for degradation but also transfer proteins and lipids to peroxisomal activation and biogenesis [92, 93]. Moreover, mitochondrial components such as BCL-2 (B-cell lymphoma 2) protein [5, 39, 40, 68, 95] and mtDNA from healthy mitochondria [21, 96, 97] can be sent to unhealthy mitochondria to recover their structure and functions, resulting in prevention of cell death [42, 98, 99].

Under pathophysiologic conditions, MDVs are the important regulator for immune response and inflammation [65, 100, 101]. During injury, mtDNA is recognized as one of the damage-associated molecular patterns (DAMPs) that can trigger pro-inflammatory response after binding to intracellular Toll-like receptors or nucleotide-binding oligomerization domain-containing protein (NOD)-like receptors [102, 103]. Additionally, mitochondrial reactive oxygen species (mtROS) has been shown to regulate proinflammatory signaling by increasing nuclear factor kappa B (NF-κB) gene expression and involving in hypoxia-inducible factor 1α (HIF1α)-induced inflammasome formation through NLRP3 (NLR family pyrin domain containing 3) [95, 104, 105]. Previous studies have also found that mitochondrial DAMPs can be released into the circulation, are recognized by pattern recognition receptors (PRRs), and promote tissue and organ injury [3, 103, 106–108]. Moreover, mitochondrial DAMPs can mediate neutrophil migration and degranulation, thereby further enhancing cellular injury and tissue inflammation [3, 103]. Many lines of evidence have shown that MDVs inhibit such inflammatory response and down-stream cascades by transferring the damaged mitochondrial components to be degraded by lysosomes and then fuse with MVB [109, 110]. Mitochondrial DAMPs in MVB are then released out as extracellular MDVs, which can inhibit pro-inflammatory activation. Moreover, MDVs-mediated antigen presentation is crucial for regulating the immune system [40, 48, 65, 111]. MDVs formation in the presence of Rab7 (a small GTPase that monitors vesicular transport to late endosomes and lysosomes), Rab9 and SNX9 (sorting nexin 9) can mediate antigen presentation after breaking down inside lysosomes by proteasome to load these mitochondrial antigens onto MHC class I molecules within endoplasmic reticulum and then transfer them to the cell surface [40, 48, 65, 111]. Therefore, MDVs are the important regulator for development, activation, differentiation and survival of diverse immune cells, including T-lymphocytes and macrophages [40, 48, 101, 112, 113].

Furthermore, MDVs can enhance anti-microbial machineries [40]. Methicillin-resistant *Staphylococcus aureus* (MRSA) infection can induce formation of MDVs containing mtROS and mitochondrial enzyme, superoxide dismutase-2 (SOD2) [114, 115]. These MDVs are then delivered to bacteria-containing phagosomes, where SOD2 can settle hydrogen peroxide activation and bacterial eradication.

## Roles of mitochondrial dysfunction in kidney stone formation

Accumulative evidence has shown the involvement of mitochondrial dysfunction and oxidative damage in KSD development [25–29, 116–119]. Mitochondria are highly abundant in renal tubular cells that require high energy for keeping their regular functions, including water reabsorption and solute transports [120–123]. Interestingly, mitochondria are enriched in epithelial cells lining renal tubular segments that have been proposed as the initial areas for kidney stone formation [124–128]. Besides, interactions of mitochondria with oxalate and CaOx crystals have been shown as the important mechanisms involved in the pathogenesis of KSD [25, 26, 116, 129–131]. Several studies have demonstrated that oxalate and/or CaOx crystals can alter mitochondrial activities and induce ROS overproduction, leading to mitochondrial dysfunction and oxidative stress [25, 26, 116, 131–134].

Mechanistically, oxidative stress-induced stimuli can activate ROS overproduction and induce mitochondrial damage [135–137]. The damaged mitochondria fail to keep membrane potential properties and, hence, release calcium ion, mtDNA, mtROS, mitochondrial matrix proteins, OMM and IMM into the cytoplasm [4, 5, 21, 35]. These mitochondrial components further induce cell death, inflammatory response and renal tubulointerstitial tissue injury [134, 138]. Such tubular cell injury has been reported to induce CaOx crystal adhesion onto the cells, leading to crystal retention inside the renal tissue that is one of the important mechanisms for kidney stone formation [134, 139–142]. Additionally, the adhered crystals can further grow and aggregate with the surrounding crystals, resulting in stone nidus formation [127, 129, 134, 141].

Additionally, the damaged mitochondria and other cellular and organellar fragments can directly bind to CaOx crystals and serve as the stone nidus for crystal nucleation, growth and aggregation, which further enhance kidney stone formation [134, 143, 144]. Moreover, the damaged mitochondria can trigger inflammatory cascade at renal interstitial area [117, 145] by recruiting numerous inflammatory cells into this area, leading to accumulation of various proinflammatory cytokines and tissue inflammation [116, 129]. Together with supersaturation of calcium phosphate, which is common in the renal interstitium, Randall’s plaque starts to form [126, 146, 147]. After erosion into the urinary space, where CaOx is frequently supersaturated, this plaque then serves as the nidus for CaOx stone to grow [25].

## Potential roles of MDVs in KSD

Several recent studies have continuously shown significant roles of urinary EVs (uEVs) in KSD [148–152]. uEVs are involved in inflammatory response and elimination of CaOx crystals, and may also serve as the composition of the stone matrix [150, 152]. Also, recent clinical studies have identified specific subtypes of uEVs as the potential biomarkers in the urine of kidney stone patients compared with healthy subjects [149, 150, 153]. Furthermore, pattern of uEVs subtypes in females with KSD (but not those derived from non-stone females) is similar to that in males with or without KSD [154]. Although MDVs have not yet been examined directly in KSD, numerous mitochondrial proteins have been identified in these uEVs. According to recent proteome and lipidome studies of MDVs [22, 46, 68], a large number of mitochondrial proteins and lipids have been identified in both MDVs and EVs [155–158]. We have also compared all of the proteins identified in EVs based on Vesiclepedia database (http://www.microvesicles.org/) with those identified in mitochondria based on The Human Protein Atlas (https://www.proteinatlas.org/). Interestingly, 244 proteins are commonly found in both EVs and mitochondria (Table 1). These findings are consistent with the data observed in recent proteome studies of EVs [46, 73]. Therefore, MDVs are expected to play similar roles as of uEVs in KSD.

**Table 1:** Summary of proteins that are found in both EVs (http://www.microvesicles.org/) and mitochondria (https://www.proteinatlas.org/)

No.	Gene symbol	Uniprot ID	Protein name(s)
1	*DECR1*	Q16698	2,4-dienoyl-CoA reductase [(3E)-enoyl-CoA-producing], mitochondrial (EC 1.3.1.124) (2,4-dienoyl-CoA reductase [NADPH]) (4-enoyl-CoA reductase [NADPH]) (Short chain dehydrogenase/reductase family 18C member 1)
2	*MRPS14*	O60783	28S ribosomal protein S14, mitochondrial (MRP-S14) (S14mt) (Mitochondrial small ribosomal subunit protein uS14m)
3	*MRPS18B*	Q9Y676	28S ribosomal protein S18b, mitochondrial (MRP-S18-b) (Mrps18-b) (S18mt-b) (28S ribosomal protein S18-2, mitochondrial) (MRP-S18-2) (Mitochondrial small ribosomal subunit protein bS18b) (Mitochondrial small ribosomal subunit protein mS40)
4	*MRPS23*	Q9Y3D9	28S ribosomal protein S23, mitochondrial (MRP-S23) (S23mt) (Mitochondrial small ribosomal subunit protein mS23)
5	*MRPS26*	Q9BYN8	28S ribosomal protein S26, mitochondrial (MRP-S26) (S26mt) (28S ribosomal protein S13, mitochondrial) (MRP-S13) (S13mt) (Mitochondrial small ribosomal subunit protein mS26)
6	*MRPS27*	Q92552	28S ribosomal protein S27, mitochondrial (MRP-S27) (S27mt) (Mitochondrial ribosomal protein S27) (Mitochondrial small ribosomal subunit protein mS27)
7	*DAP3*	P51398	28S ribosomal protein S29, mitochondrial (MRP-S29) (S29mt) (Death-associated protein 3) (DAP-3) (Ionizing radiation resistance conferring protein) (Mitochondrial small ribosomal subunit protein mS29)
8	*MRPS31*	Q92665	28S ribosomal protein S31, mitochondrial (MRP-S31) (S31mt) (Imogen 38) (Mitochondrial small ribosomal subunit protein mS31)
9	*MRPS35*	P82673	28S ribosomal protein S35, mitochondrial (MRP-S35) (S35mt) (28S ribosomal protein S28, mitochondrial) (MRP-S28) (S28mt) (Mitochondrial small ribosomal subunit protein mS35)
10	*BCKDHB*	P21953	2-oxoisovalerate dehydrogenase subunit beta, mitochondrial (EC 1.2.4.4) (Branched-chain alpha-keto acid dehydrogenase E1 component beta chain) (BCKDE1B) (BCKDH E1-beta)
11	*MRPL2*	Q5T653	39S ribosomal protein L2, mitochondrial (L2mt) (MRP-L2) (Mitochondrial large ribosomal subunit protein uL2m)
12	*MRPL21*	Q7Z2W9	39S ribosomal protein L21, mitochondrial (L21mt) (MRP-L21) (Mitochondrial large ribosomal subunit protein bL21m)
13	*MRPL23*	Q16540	39S ribosomal protein L23, mitochondrial (L23mt) (MRP-L23) (L23 mitochondrial-related protein) (Mitochondrial large ribosomal subunit protein uL23m) (Ribosomal protein L23-like)
14	*MRPL36*	Q9P0J6	39S ribosomal protein L36, mitochondrial (L36mt) (MRP-L36) (BRCA1-interacting protein 1) (Mitochondrial large ribosomal subunit protein bL36m)
15	*MRPL40*	Q9NQ50	39S ribosomal protein L40, mitochondrial (L40mt) (MRP-L40) (Mitochondrial large ribosomal subunit protein mL40) (Nuclear localization signal-containing protein deleted in velocardiofacial syndrome) (Up-regulated in metastasis)
16	*MRPL43*	Q8N983	39S ribosomal protein L43, mitochondrial (L43mt) (MRP-L43) (Mitochondrial large ribosomal subunit protein mL43) (Mitochondrial ribosomal protein bMRP36a)
17	*MRPL44*	Q9H9J2	39S ribosomal protein L44, mitochondrial (L44mt) (MRP-L44) (EC 3.1.26.-) (Mitochondrial large ribosomal subunit protein mL44)
18	*MRPL46*	Q9H2W6	39S ribosomal protein L46, mitochondrial (L46mt) (MRP-L46) (Mitochondrial large ribosomal subunit protein mL46) (P2ECSL)
19	*MRPL52*	Q86TS9	39S ribosomal protein L52, mitochondrial (L52mt) (MRP-L52) (Mitochondrial large ribosomal subunit protein mL52)
20	*MPST*	P25325	3-mercaptopyruvate sulfurtransferase (MST) (EC 2.8.1.2)
21	*HPDL*	Q96IR7	4-hydroxyphenylpyruvate dioxygenase-like protein (HPD-like protein) (EC 1.13.-.-) (Glyoxalase domain-containing protein 1)
22	*NT5DC3*	Q86UY8	5′-nucleotidase domain-containing protein 3 (EC 3.1.3.-) (GRP94-neighboring nucleotidase)
23	*RPL7L1*	Q6DKI1	60S ribosomal protein L7-like 1 (Large ribosomal subunit protein uL30-like 1)
24	*ADAMTS16*	Q8TE57	A disintegrin and metalloproteinase with thrombospondin motifs 16 (ADAM-TS 16) (ADAM-TS16) (ADAMTS-16) (EC 3.4.24.-)
25	*SMPDL3A*	Q92484	Acid sphingomyelinase-like phosphodiesterase 3a (ASM-like phosphodiesterase 3a) (EC 3.1.4.-)
26	*NDUFAB1*	O14561	Acyl carrier protein, mitochondrial (ACP) (CI-SDAP) (NADH-ubiquinone oxidoreductase 9.6 kDa subunit)
27	*AGK*	Q53H12	Acylglycerol kinase, mitochondrial (hAGK) (EC 2.7.1.107) (EC 2.7.1.138) (EC 2.7.1.94) (Multiple substrate lipid kinase) (HsMuLK) (MuLK) (Multi-substrate lipid kinase)
28	*FAHD1*	Q6P587	Acylpyruvase FAHD1, mitochondrial (EC 3.7.1.5) (Fumarylacetoacetate hydrolase domain-containing protein 1) (FAH domain-containing protein 1) (Oxaloacetate decarboxylase) (OAA decarboxylase) (EC 4.1.1.112) (YisK-like protein)
29	*NUDT9*	Q9BW91	ADP-ribose pyrophosphatase, mitochondrial (EC 3.6.1.13) (ADP-ribose diphosphatase) (ADP-ribose phosphohydrolase) (Adenosine diphosphoribose pyrophosphatase) (ADPR-PPase) (Nucleoside diphosphate-linked moiety X motif 9) (Nudix motif 9)
30	*AARS2*	Q5JTZ9	Alanine–tRNA ligase, mitochondrial (EC 6.1.1.7) (Alanyl-tRNA synthetase) (AlaRS)
31	*AARS2*	Q5JTZ9	Alanine–tRNA ligase, mitochondrial
32	*ALDH1B1*	P30837	Aldehyde dehydrogenase X, mitochondrial (EC 1.2.1.3) (Aldehyde dehydrogenase 5) (Aldehyde dehydrogenase family 1 member B1)
33	*ALDH7A1*	P49419	Alpha-aminoadipic semialdehyde dehydrogenase (Alpha-AASA dehydrogenase) (EC 1.2.1.31) (Aldehyde dehydrogenase family 7 member A1) (EC 1.2.1.3) (Antiquitin-1) (Betaine aldehyde dehydrogenase) (EC 1.2.1.8) (Delta1-piperideine-6-carboxylate dehydrogenase) (P6c dehydrogenase)
34	*AASS*	Q9UDR5	Alpha-aminoadipic semialdehyde synthase, mitochondrial (LKR/SDH) [Includes: Lysine ketoglutarate reductase (LKR) (LOR) (EC 1.5.1.8); Saccharopine dehydrogenase (SDH) (EC 1.5.1.9)]
35	*MAOA*	P21397	Amine oxidase [flavin-containing] A (EC 1.4.3.4) (Monoamine oxidase type A) (MAO-A)
36	*ADGB*	Q8N7X0	Androglobin (Calpain-7-like protein)
37	*ANKRD34B*	A5PLL1	Ankyrin repeat domain-containing protein 34B
38	*ARMCX1*	Q9P291	Armadillo repeat-containing X-linked protein 1 (ARM protein lost in epithelial cancers on chromosome X 1) (Protein ALEX1)
39	*ARMCX2*	Q7L311	Armadillo repeat-containing X-linked protein 2 (ARM protein lost in epithelial cancers on chromosome X 2) (Protein ALEX2)
40	*DARS2*	Q6PI48	Aspartate–tRNA ligase, mitochondrial (EC 6.1.1.12) (Aspartyl-tRNA synthetase) (AspRS)
41	*ATP5MF*	P56134	ATP synthase subunit f, mitochondrial (ATP synthase membrane subunit f)
42	*PFKL*	P17858	ATP-dependent 6-phosphofructokinase, liver type (ATP-PFK) (PFK-L) (EC 2.7.1.11) (6-phosphofructokinase type B) (Phosphofructo-1-kinase isozyme B) (PFK-B) (Phosphohexokinase)
43	*CLPX*	O76031	ATP-dependent Clp protease ATP-binding subunit clpX-like, mitochondrial
44	*DHX30*	Q7L2E3	ATP-dependent RNA helicase DHX30 (EC 3.6.4.13) (DEAH box protein 30)
45	*YME1L1*	Q96TA2	ATP-dependent zinc metalloprotease YME1L1 (EC 3.4.24.-) (ATP-dependent metalloprotease FtsH1) (Meg-4) (Presenilin-associated metalloprotease) (PAMP) (YME1-like protein 1)
46	*ATRNL1*	Q5VV63	Attractin-like protein 1
47	*AURKAIP1*	Q9NWT8	Aurora kinase A-interacting protein (AURKA-interacting protein) (28S ribosomal protein S38, mitochondrial) (MRP-S38) (Mitochondrial small ribosomal subunit protein mS38)
48	*CD72*	P21854	B-cell differentiation antigen CD72 (Lyb-2) (CD antigen CD72)
49	*BOLA3*	Q53S33	BolA-like protein 3
50	*BDNF*	P23560	Brain-derived neurotrophic factor (BDNF) (Abrineurin) [Cleaved into: BDNF precursor form (ProBDNF)]
51	*BRI3BP*	Q8WY22	BRI3-binding protein (I3-binding protein) (Cervical cancer 1 proto-oncogene-binding protein KG19) (HCCRBP-1)
52	*KCTD6*	Q8NC69	BTB/POZ domain-containing protein KCTD6 (KCASH3 protein) (Potassium channel tetramerization domain-containing protein 6)
53	*CALHM2*	Q9HA72	Calcium homeostasis modulator protein 2 (Protein FAM26B)
54	*CASQ1*	P31415	Calsequestrin-1 (Calmitine) (Calsequestrin, skeletal muscle isoform)
55	*CPT2*	P23786	Carnitine *O*-palmitoyltransferase 2, mitochondrial (EC 2.3.1.21) (Carnitine palmitoyltransferase II) (CPT II)
56	*CASP3*	P42574	Caspase-3 (CASP-3) (EC 3.4.22.56) (Apopain) (Cysteine protease CPP32) (CPP-32) (Protein Yama) (SREBP cleavage activity 1) (SCA-1) [Cleaved into: Caspase-3 subunit p17; Caspase-3 subunit p12]
57	*SLC44A1*	Q8WWI5	Choline transporter-like protein 1 (CDw92) (Solute carrier family 44 member 1) (CD antigen CD92)
58	*CBX6*	O95503	Chromobox protein homolog 6
59	*C21orf2*	O43822	Cilia- and flagella-associated protein 410 (C21orf-HUMF09G8.5) (Leucine-rich repeat-containing protein 76) (YF5/A2)
60	*CNKSR3*	Q6P9H4	Connector enhancer of kinase suppressor of ras 3 (Connector enhancer of KSR 3) (CNK homolog protein 3) (CNK3) (CNKSR family member 3) (Maguin-like protein)
61	*ATG4D*	Q86TL0	Cysteine protease ATG4D (EC 3.4.22.-) (AUT-like 4 cysteine endopeptidase) (Autophagin-4) (Autophagy-related cysteine endopeptidase 4) (Autophagy-related protein 4 homolog D) [Cleaved into: Cysteine protease ATG4D, mitochondrial]
62	*COX7A2L*	O14548	Cytochrome c oxidase subunit 7A-related protein, mitochondrial (COX7a-related protein) (Cytochrome c oxidase subunit VIIa-related protein) (EB1)
63	*CYC1*	P08574	Cytochrome c1, heme protein, mitochondrial (EC 7.1.1.8) (Complex III subunit 4) (Complex III subunit IV) (Cytochrome b-c1 complex subunit 4) (Ubiquinol-cytochrome-c reductase complex cytochrome c1 subunit) (Cytochrome c-1)
64	*DYNC2H1*	Q8NCM8	Cytoplasmic dynein 2 heavy chain 1 (Cytoplasmic dynein 2 heavy chain) (Dynein cytoplasmic heavy chain 2) (Dynein heavy chain 11) (hDHC11) (Dynein heavy chain isotype 1B)
65	*DCAF15*	Q66K64	DDB1- and CUL4-associated factor 15
66	*DHRS2*	Q13268	Dehydrogenase/reductase SDR family member 2, mitochondrial (EC 1.1.1.-) (Dicarbonyl reductase HEP27) (Protein D) (Short chain dehydrogenase/reductase family 25C member 1)
67	*DHRS7*	Q9Y394	Dehydrogenase/reductase SDR family member 7 (EC 1.1.-.-) (Retinal short-chain dehydrogenase/reductase 4) (retSDR4) (Short chain dehydrogenase/reductase family 34C member 1)
68	*DEPTOR*	Q8TB45	DEP domain-containing mTOR-interacting protein (DEP domain-containing protein 6)
69	*DIABLO*	Q9NR28	Diablo homolog, mitochondrial
70	*DIABLO*	Q9NR28	Diablo homolog, mitochondrial (Direct IAP-binding protein with low pI) (Second mitochondria-derived activator of caspase) (Smac)
71	*DLD*	P09622	Dihydrolipoyl dehydrogenase, mitochondrial (EC 1.8.1.4) (Dihydrolipoamide dehydrogenase) (Glycine cleavage system L protein)
72	*DLST*	P36957	Dihydrolipoyllysine-residue succinyltransferase component of 2-oxoglutarate dehydrogenase complex, mitochondrial (EC 2.3.1.61) (2-oxoglutarate dehydrogenase complex component E2) (OGDC-E2) (Dihydrolipoamide succinyltransferase component of 2-oxoglutarate dehydrogenase complex) (E2K)
73	*DHODH*	Q02127	Dihydroorotate dehydrogenase (quinone), mitochondrial (DHOdehase) (EC 1.3.5.2) (Dihydroorotate oxidase)
74	*DNAJA3*	Q96EY1	DnaJ homolog subfamily A member 3, mitochondrial (DnaJ protein Tid-1) (hTid-1) (Hepatocellular carcinoma-associated antigen 57) (Tumorous imaginal discs protein Tid56 homolog)
75	*DNLZ*	Q5SXM8	DNL-type zinc finger protein (Hsp70-escort protein 1) (HEP1) (mtHsp70-escort protein)
76	*DMRTA2*	Q96SC8	Doublesex- and mab-3-related transcription factor A2 (Doublesex- and mab-3-related transcription factor 5)
77	*OPA1*	O60313	Dynamin-like 120 kDa protein, mitochondrial (EC 3.6.5.5) (Optic atrophy protein 1) [Cleaved into: Dynamin-like 120 kDa protein, form S1]
78	*RNF115*	Q9Y4L5	E3 ubiquitin-protein ligase RNF115 (EC 2.3.2.27) (RING finger protein 115) (RING-type E3 ubiquitin transferase RNF115) (Rabring 7) (Zinc finger protein 364)
79	*SIAH1*	Q8IUQ4	E3 ubiquitin-protein ligase SIAH1 (EC 2.3.2.27) (RING-type E3 ubiquitin transferase SIAH1) (Seven in absentia homolog 1) (Siah-1) (Siah-1a)
80	*EML6*	Q6ZMW3	Echinoderm microtubule-associated protein-like 6 (EMAP-6) (Echinoderm microtubule-associated protein-like 5-like)
81	*GFM1*	Q96RP9	Elongation factor G, mitochondrial (EF-Gmt) (Elongation factor G 1, mitochondrial) (mEF-G 1) (Elongation factor G1) (hEFG1)
82	*TSFM*	P43897	Elongation factor Ts, mitochondrial (EF-Ts) (EF-TsMt)
83	*ECI2*	O75521	Enoyl-CoA delta isomerase 2 (EC 5.3.3.8) (DRS-1) (Delta(3),delta(2)-enoyl-CoA isomerase) (D3,D2-enoyl-CoA isomerase) (Diazepam-binding inhibitor-related protein 1) (DBI-related protein 1) (Dodecenoyl-CoA isomerase) (Hepatocellular carcinoma-associated antigen 88) (Peroxisomal 3,2-trans-enoyl-CoA isomerase) (pECI) (Renal carcinoma antigen NY-REN-1)
84	*EIF4E2*	O60573	Eukaryotic translation initiation factor 4E type 2 (eIF-4E type 2) (eIF4E type 2) (Eukaryotic translation initiation factor 4E homologous protein) (Eukaryotic translation initiation factor 4E-like 3) (eIF4E-like protein 4E-LP) (mRNA cap-binding protein 4EHP) (h4EHP) (mRNA cap-binding protein type 3)
85	*SLC1A3*	P43003	Excitatory amino acid transporter 1 (Sodium-dependent glutamate/aspartate transporter 1) (GLAST-1) (Solute carrier family 1 member 3)
86	*EXOC3*	O60645	Exocyst complex component 3 (Exocyst complex component Sec6)
87	*EXD2*	Q9NVH0	Exonuclease 3'-5' domain-containing protein 2 (EC 3.1.11.1) (3'-5' exoribonuclease EXD2) (EC 3.1.13.-) (Exonuclease 3'-5' domain-like-containing protein 2)
88	*FASTK*	Q14296	Fas-activated serine/threonine kinase (FAST kinase) (EC 2.7.11.8)
89	*FSTL4*	Q6MZW2	Follistatin-related protein 4 (Follistatin-like protein 4)
90	*FOXN4*	Q96NZ1	Forkhead box protein N4
91	*FXN*	Q16595	Frataxin, mitochondrial (EC 1.16.3.1) (Friedreich ataxia protein) (Fxn) [Cleaved into: Frataxin intermediate form (i-FXN); Frataxin(56–210) (m56-FXN); Frataxin(78–210) (d-FXN) (m78-FXN); Frataxin mature form (Frataxin(81–210)) (m81-FXN)]
92	*LGALS2*	P05162	Galectin-2 (Gal-2) (Beta-galactoside-binding lectin L-14-II) (HL14) (Lactose-binding lectin 2) (S-Lac lectin 2)
93	*GDAP1*	Q8TB36	Ganglioside-induced differentiation-associated protein 1 (GDAP1)
94	*GSE1*	Q14687	Genetic suppressor element 1
95	*FP565260.6*	A0A0B4J2D5	Glutamine amidotransferase-like class 1 domain-containing protein 3B, mitochondrial (Keio novel protein-I) (KNP-I) (Protein GT335) (Protein HES1)
96	*GLDC*	P23378	Glycine dehydrogenase (decarboxylating), mitochondrial (EC 1.4.4.2) (Glycine cleavage system P protein) (Glycine decarboxylase) (Glycine dehydrogenase (aminomethyl-transferring))
97	*GADD45GIP1*	Q8TAE8	Growth arrest and DNA damage-inducible proteins-interacting protein 1 (39S ribosomal protein L59, mitochondrial) (MRP-L59) (CKII beta-associating protein) (CR6-interacting factor 1) (CRIF1) (Mitochondrial large ribosomal subunit protein mL64) (Papillomavirus L2-interacting nuclear protein 1) (PLINP) (PLINP-1) (p53-responsive gene 6 protein)
98	*AC093155.3*	Q7LGA3	Heparan sulfate 2-O-sulfotransferase 1
99	*HS2ST1*	Q7LGA3	Heparan sulfate 2-O-sulfotransferase 1 (2-O-sulfotransferase) (2OST) (EC 2.8.2.-)
100	*HHIPL2*	Q6UWX4	HHIP-like protein 2
101	*HIGD1A*	Q9Y241	HIG1 domain family member 1A, mitochondrial (Hypoxia-inducible gene 1 protein) (RCF1 homolog A) (RCF1a)
102	*HIGD2A*	Q9BW72	HIG1 domain family member 2A, mitochondrial (RCF1 homolog B) (RCF1b)
103	*HINT3*	Q9NQE9	Histidine triad nucleotide-binding protein 3 (HINT-3) (EC 3.-.-.-)
104	*NSD3*	Q9BZ95	Histone-lysine N-methyltransferase NSD3 (EC 2.1.1.370) (EC 2.1.1.371) (Nuclear SET domain-containing protein 3) (Protein whistle) (WHSC1-like 1 isoform 9 with methyltransferase activity to lysine) (Wolf-Hirschhorn syndrome candidate 1-like protein 1) (WHSC1-like protein 1)
105	*HCFC1*	P51610	Host cell factor 1 (HCF) (HCF-1) (C1 factor) (CFF) (VCAF) (VP16 accessory protein) [Cleaved into: HCF N-terminal chain 1; HCF N-terminal chain 2; HCF N-terminal chain 3; HCF N-terminal chain 4; HCF N-terminal chain 5; HCF N-terminal chain 6; HCF C-terminal chain 1; HCF C-terminal chain 2; HCF C-terminal chain 3; HCF C-terminal chain 4; HCF C-terminal chain 5; HCF C-terminal chain 6]
106	*HSDL1*	Q3SXM5	Inactive hydroxysteroid dehydrogenase-like protein 1 (Short chain dehydrogenase/reductase family 12C member 3)
107	*PLD5*	Q8N7P1	Inactive phospholipase D5 (Inactive PLD 5) (Inactive choline phosphatase 5) (Inactive phosphatidylcholine-hydrolyzing phospholipase D5) (PLDc)
108	*ITGB5*	P18084	Integrin beta-5
109	*ICAM3*	P32942	Intercellular adhesion molecule 3 (ICAM-3) (CDw50) (ICAM-R) (CD antigen CD50)
110	*ILF3*	Q12906	Interleukin enhancer-binding factor 3 (Double-stranded RNA-binding protein 76) (DRBP76) (M-phase phosphoprotein 4) (MPP4) (Nuclear factor associated with dsRNA) (NFAR) (Nuclear factor of activated T-cells 90 kDa) (NF-AT-90) (Translational control protein 80) (TCP80)
111	*IFT27*	Q9BW83	Intraflagellar transport protein 27 homolog (Putative GTP-binding protein RAY-like) (Rab-like protein 4)
112	*ISOC2*	Q96AB3	Isochorismatase domain-containing protein 2
113	*IDH3G*	P51553	Isocitrate dehydrogenase [NAD] subunit gamma, mitochondrial (Isocitric dehydrogenase subunit gamma) (NAD( +)-specific ICDH subunit gamma)
114	*IVD*	P26440	Isovaleryl-CoA dehydrogenase, mitochondrial (IVD) (EC 1.3.8.4) (Butyryl-CoA dehydrogenase) (EC 1.3.8.1)
115	*KLHL29*	Q96CT2	Kelch-like protein 29 (Kelch repeat and BTB domain-containing protein 9)
116	*KLC4*	Q9NSK0	Kinesin light chain 4 (KLC 4) (Kinesin-like protein 8)
117	*LRRD1*	A4D1F6	Leucine-rich repeat and death domain-containing protein 1
118	*LIAS*	O43766	Lipoyl synthase, mitochondrial (EC 2.8.1.8) (Lipoate synthase) (LS) (Lip-syn) (Lipoic acid synthase)
119	*LONP1*	P36776	Lon protease homolog, mitochondrial (EC 3.4.21.53) (LONHs) (Lon protease-like protein) (LONP) (Mitochondrial ATP-dependent protease Lon) (Serine protease 15)
120	*ACSL5*	Q9ULC5	Long-chain-fatty-acid–CoA ligase 5 (EC 6.2.1.3) (Arachidonate–CoA ligase) (EC 6.2.1.15) (Long-chain acyl-CoA synthetase 5) (LACS 5)
121	*LRP12*	Q9Y561	Low-density lipoprotein receptor-related protein 12 (LDLR-related protein 12) (LRP-12) (Suppressor of tumorigenicity 7 protein)
122	*LRP4*	O75096	Low-density lipoprotein receptor-related protein 4 (LRP-4) (Multiple epidermal growth factor-like domains 7)
123	*MFSD12*	Q6NUT3	Major facilitator superfamily domain-containing protein 12
124	*XK*	P51811	Membrane transport protein XK (Kell complex 37 kDa component) (Kx antigen) (XK-related protein 1)
125	*MBLAC2*	Q68D91	Metallo-beta-lactamase domain-containing protein 2 (EC 3.-.-.-)
126	*MTX2*	O75431	Metaxin-2 (Mitochondrial outer membrane import complex protein 2)
127	*C19orf70*	Q5XKP0	MICOS complex subunit MIC13 (Protein P117)
128	*APOO*	Q9BUR5	MICOS complex subunit MIC26 (Apolipoprotein O) (MICOS complex subunit MIC23) (Protein FAM121B)
129	*MGST1*	P10620	Microsomal glutathione S-transferase 1 (Microsomal GST-1) (EC 2.5.1.18) (Microsomal GST-I)
130	*SLC25A10*	Q9UBX3	Mitochondrial dicarboxylate carrier (Solute carrier family 25 member 10)
131	*SLC25A22*	Q9H936	Mitochondrial glutamate carrier 1 (GC-1) (Glutamate/H( +) symporter 1) (Solute carrier family 25 member 22)
132	*SLC25A18*	Q9H1K4	Mitochondrial glutamate carrier 2 (GC-2) (Glutamate/H( +) symporter 2) (Solute carrier family 25 member 18)
133	*TIMM13*	Q9Y5L4	Mitochondrial import inner membrane translocase subunit Tim13
134	*PAM16*	Q9Y3D7	Mitochondrial import inner membrane translocase subunit TIM16 (Mitochondria-associated granulocyte macrophage CSF-signaling molecule) (Presequence translocated-associated motor subunit PAM16)
135	*TIMM50*	Q3ZCQ8	Mitochondrial import inner membrane translocase subunit TIM50
136	*TOMM40*	O96008	Mitochondrial import receptor subunit TOM40 homolog (Protein Haymaker) (Translocase of outer membrane 40 kDa subunit homolog) (p38.5)
137	*CCDC51*	Q96ER9	Mitochondrial potassium channel (MITOK) (Coiled-coil domain-containing protein 51)
138	*ABCB8*	Q9NUT2	Mitochondrial potassium channel ATP-binding subunit (ATP-binding cassette sub-family B member 8, mitochondrial) (ABCB8) (Mitochondrial ATP-binding cassette 1) (M-ABC1) (Mitochondrial sulfonylurea-receptor) (MITOSUR)
139	*KIAA0391*	O15091	Mitochondrial ribonuclease P catalytic subunit (EC 3.1.26.5) (Mitochondrial ribonuclease P protein 3) (Mitochondrial RNase P protein 3) (Protein only RNase P catalytic subunit)
140	*SLC25A37*	Q9NYZ2	Mitoferrin-1 (Mitochondrial iron transporter 1) (Mitochondrial solute carrier protein) (Solute carrier family 25 member 37)
141	*MOCOS*	Q96EN8	Molybdenum cofactor sulfurase (MCS) (MOS) (MoCo sulfurase) (hMCS) (EC 2.8.1.9) (Molybdenum cofactor sulfurtransferase)
142	*MORN1*	Q5T089	MORN repeat-containing protein 1
143	*MYL3*	P08590	Myosin light chain 3 (Cardiac myosin light chain 1) (CMLC1) (Myosin light chain 1, slow-twitch muscle B/ventricular isoform) (MLC1SB) (Ventricular myosin alkali light chain) (Ventricular myosin light chain 1) (VLCl) (Ventricular/slow twitch myosin alkali light chain) (MLC-lV/sb)
144	*B3GNT4*	Q9C0J1	N-acetyllactosaminide beta-1,3-N-acetylglucosaminyltransferase 4 (EC 2.4.1.149) (UDP-GlcNAc:betaGal beta-1,3-N-acetylglucosaminyltransferase 4) (BGnT-4) (Beta-1,3-Gn-T4) (Beta-1,3-N-acetylglucosaminyltransferase 4) (Beta3Gn-T4)
145	*NNT*	Q13423	NAD(P) transhydrogenase, mitochondrial (EC 7.1.1.1) (Nicotinamide nucleotide transhydrogenase) (Pyridine nucleotide transhydrogenase)
146	*NDUFA12*	Q9UI09	NADH dehydrogenase [ubiquinone] 1 alpha subcomplex subunit 12 (13 kDa differentiation-associated protein) (Complex I-B17.2) (CI-B17.2) (CIB17.2) (NADH-ubiquinone oxidoreductase subunit B17.2)
147	*NDUFA9*	Q16795	NADH dehydrogenase [ubiquinone] 1 alpha subcomplex subunit 9, mitochondrial (Complex I-39kD) (CI-39kD) (NADH-ubiquinone oxidoreductase 39 kDa subunit)
148	*NDUFB1*	O75438	NADH dehydrogenase [ubiquinone] 1 beta subcomplex subunit 1 (Complex I-MNLL) (CI-MNLL) (NADH-ubiquinone oxidoreductase MNLL subunit)
149	*NDUFB4*	O95168	NADH dehydrogenase [ubiquinone] 1 beta subcomplex subunit 4 (Complex I-B15) (CI-B15) (NADH-ubiquinone oxidoreductase B15 subunit)
150	*NDUFB5*	O43674	NADH dehydrogenase [ubiquinone] 1 beta subcomplex subunit 5, mitochondrial (Complex I-SGDH) (CI-SGDH) (NADH-ubiquinone oxidoreductase SGDH subunit)
151	*NDUFV1*	P49821	NADH dehydrogenase [ubiquinone] flavoprotein 1, mitochondrial (EC 7.1.1.2) (Complex I-51kD) (CI-51kD) (NADH dehydrogenase flavoprotein 1) (NADH-ubiquinone oxidoreductase 51 kDa subunit)
152	*NDUFV2*	P19404	NADH dehydrogenase [ubiquinone] flavoprotein 2, mitochondrial (EC 7.1.1.2) (NADH-ubiquinone oxidoreductase 24 kDa subunit)
153	*NDUFS3*	O75489	NADH dehydrogenase [ubiquinone] iron-sulfur protein 3, mitochondrial (EC 7.1.1.2) (Complex I-30kD) (CI-30kD) (NADH-ubiquinone oxidoreductase 30 kDa subunit)
154	*CYB5R1*	Q9UHQ9	NADH-cytochrome b5 reductase 1 (b5R.1) (EC 1.6.2.2) (Humb5R2) (NAD(P)H:quinone oxidoreductase type 3 polypeptide A2)
155	*SLC11A1*	P49279	Natural resistance-associated macrophage protein 1 (NRAMP 1) (Solute carrier family 11 member 1)
156	*NGRN*	Q9NPE2	Neugrin (Mesenchymal stem cell protein DSC92) (Neurite outgrowth-associated protein) (Spinal cord-derived protein FI58G)
157	*NGDN*	Q8NEJ9	Neuroguidin (Centromere accumulated nuclear protein 1) (CANu1) (EIF4E-binding protein)
158	*SLC3A1*	Q07837	Neutral and basic amino acid transport protein rBAT (NBAT) (D2h) (Solute carrier family 3 member 1) (b(0, +)-type amino acid transport protein)
159	*NLRX1*	Q86UT6	NLR family member X1 (Caterpiller protein 11.3) (CLR11.3) (Nucleotide-binding oligomerization domain protein 26) (Nucleotide-binding oligomerization domain protein 5) (Nucleotide-binding oligomerization domain protein 9)
160	*NACC2*	Q96BF6	Nucleus accumbens-associated protein 2 (NAC-2) (BTB/POZ domain-containing protein 14A) (Repressor with BTB domain and BEN domain)
161	*REXO2*	Q9Y3B8	Oligoribonuclease, mitochondrial (EC 3.1.-.-) (RNA exonuclease 2 homolog) (Small fragment nuclease)
162	*OAT*	P04181	Ornithine aminotransferase, mitochondrial (EC 2.6.1.13) (Ornithine delta-aminotransferase) (Ornithine–oxo-acid aminotransferase) [Cleaved into: Ornithine aminotransferase, hepatic form; Ornithine aminotransferase, renal form]
163	*PAX9*	P55771	Paired box protein Pax-9
164	*PDF*	Q9HBH1	Peptide deformylase, mitochondrial (EC 3.5.1.88) (Polypeptide deformylase)
165	*FKBP8*	Q14318	Peptidyl-prolyl cis–trans isomerase FKBP8 (PPIase FKBP8) (EC 5.2.1.8) (38 kDa FK506-binding protein) (38 kDa FKBP) (FKBP-38) (hFKBP38) (FK506-binding protein 8) (FKBP-8) (FKBPR38) (Rotamase)
166	*MRPL58*	Q14197	Peptidyl-tRNA hydrolase ICT1, mitochondrial (EC 3.1.1.29) (39S ribosomal protein L58, mitochondrial) (MRP-L58) (Digestion substraction 1) (DS-1) (Immature colon carcinoma transcript 1 protein) (Mitochondrial large ribosomal subunit protein mL62)
167	*GPX4*	P36969	Phospholipid hydroperoxide glutathione peroxidase (PHGPx) (EC 1.11.1.12) (Glutathione peroxidase 4) (GPx-4) (GSHPx-4)
168	*PIWIL4*	Q7Z3Z4	Piwi-like protein 4
169	*PCBP3*	P57721	Poly(rC)-binding protein 3 (Alpha-CP3) (PCBP3-overlapping transcript) (PCBP3-overlapping transcript 1)
170	*PNPT1*	Q8TCS8	Polyribonucleotide nucleotidyltransferase 1, mitochondrial (EC 2.7.7.8) (3'-5' RNA exonuclease OLD35) (PNPase old-35) (Polynucleotide phosphorylase 1) (PNPase 1) (Polynucleotide phosphorylase-like protein)
171	*KCNH3*	Q9ULD8	Potassium voltage-gated channel subfamily H member 3 (Brain-specific eag-like channel 1) (BEC1) (Ether-a-go-go-like potassium channel 2) (ELK channel 2) (ELK2) (Voltage-gated potassium channel subunit Kv12.2)
172	*PCYOX1L*	Q8NBM8	Prenylcysteine oxidase-like (EC 1.8.3.-)
173	*DPY19L2*	Q6NUT2	Probable C-mannosyltransferase DPY19L2 (EC 2.4.1.-) (Dpy-19-like protein 2) (Protein dpy-19 homolog 2)
174	*CARS2*	Q9HA77	Probable cysteine–tRNA ligase, mitochondrial (EC 6.1.1.16) (Cysteinyl-tRNA synthetase) (CysRS)
175	*EARS2*	Q5JPH6	Probable glutamate–tRNA ligase, mitochondrial (EC 6.1.1.17) (Glutamyl-tRNA synthetase) (GluRS)
176	*LARS2*	Q15031	Probable leucine–tRNA ligase, mitochondrial (EC 6.1.1.4) (Leucyl-tRNA synthetase) (LeuRS)
177	*PSTPIP2*	Q9H939	Proline-serine-threonine phosphatase-interacting protein 2 (PEST phosphatase-interacting protein 2)
178	*PSMG4*	Q5JS54	Proteasome assembly chaperone 4 (PAC-4) (hPAC4)
179	*SELENOO*	Q9BVL4	Protein adenylyltransferase SelO, mitochondrial (EC 2.7.7.-) (EC 2.7.7.n1) (Selenoprotein O) (SelO)
180	*ATOH1*	Q92858	Protein atonal homolog 1 (Class A basic helix-loop-helix protein 14) (bHLHa14) (Helix-loop-helix protein hATH-1) (hATH1)
181	*FAM171B*	Q6P995	Protein FAM171B
182	*FAM181B*	A6NEQ2	Protein FAM181B
183	*FAM234A*	Q9H0X4	Protein FAM234A (Protein ITFG3)
184	*FAM83F*	Q8NEG4	Protein FAM83F
185	*JARID2*	Q92833	Protein Jumonji (Jumonji/ARID domain-containing protein 2)
186	*CCDC58*	Q4VC31	Protein MIX23 (Coiled-coil domain-containing protein 58)
187	*SCO2*	O43819	Protein SCO2 homolog, mitochondrial
188	*SCO2*	O43819	Protein SCO2 homolog, mitochondrial
189	*PCMTD2*	Q9NV79	Protein-l-isoaspartate *O*-methyltransferase domain-containing protein 2
190	*PPOX*	P50336	Protoporphyrinogen oxidase (PPO) (EC 1.3.3.4)
191	*PDK3*	Q15120	Pyruvate dehydrogenase (acetyl-transferring)] kinase isozyme 3, mitochondrial (EC 2.7.11.2) (Pyruvate dehydrogenase kinase isoform 3
192	*PDHB*	P11177	Pyruvate dehydrogenase E1 component subunit beta, mitochondrial (PDHE1-B) (EC 1.2.4.1)
193	*RAB38*	P57729	Ras-related protein Rab-38 (Melanoma antigen NY-MEL-1)
194	*RIMS3*	Q9UJD0	Regulating synaptic membrane exocytosis protein 3 (Nim3) (RIM3 gamma) (Rab-3-interacting molecule 3) (RIM 3)
195	*RMDN3*	Q96TC7	Regulator of microtubule dynamics protein 3 (RMD-3) (hRMD-3) (Cerebral protein 10) (Protein FAM82A2) (Protein FAM82C) (Protein tyrosine phosphatase-interacting protein 51) (TCPTP-interacting protein 51)
196	*RTKN*	Q9BST9	Rhotekin
197	*RPS6KA6*	Q9UK32	Ribosomal protein S6 kinase alpha-6 (S6K-alpha-6) (EC 2.7.11.1) (90 kDa ribosomal protein S6 kinase 6) (p90-RSK 6) (p90RSK6) (Ribosomal S6 kinase 4) (RSK-4) (pp90RSK4)
198	*GFM2*	Q969S9	Ribosome-releasing factor 2, mitochondrial (RRF2mt) (Elongation factor G 2, mitochondrial) (EF-G2mt) (mEF-G 2) (Elongation factor G2) (hEFG2)
199	*SHMT2*	P34897	Serine hydroxymethyltransferase, mitochondrial (SHMT) (EC 2.1.2.1) (Glycine hydroxymethyltransferase) (Serine methylase)
200	*ANKRD44*	Q8N8A2	Serine/threonine-protein phosphatase 6 regulatory ankyrin repeat subunit B (PP6-ARS-B) (Serine/threonine-protein phosphatase 6 regulatory subunit ARS-B) (Ankyrin repeat domain-containing protein 44)
201	*ANKRD52*	Q8NB46	Serine/threonine-protein phosphatase 6 regulatory ankyrin repeat subunit C (PP6-ARS-C) (Serine/threonine-protein phosphatase 6 regulatory subunit ARS-C) (Ankyrin repeat domain-containing protein 52)
202	*SARS2*	Q9NP81	Serine–tRNA ligase, mitochondrial (EC 6.1.1.11) (SerRSmt) (Seryl-tRNA synthetase) (SerRS) (Seryl-tRNA(Ser/Sec) synthetase)
203	*DHRS3*	O75911	Short-chain dehydrogenase/reductase 3 (EC 1.1.1.300) (DD83.1) (Retinal short-chain dehydrogenase/reductase 1) (retSDR1) (Retinol dehydrogenase 17) (Short chain dehydrogenase/reductase family 16C member 1)
204	*ACADS*	P16219	Short-chain specific acyl-CoA dehydrogenase, mitochondrial (SCAD) (EC 1.3.8.1) (Butyryl-CoA dehydrogenase)
205	*SLC6A13*	Q9NSD5	Sodium- and chloride-dependent GABA transporter 2 (GAT-2) (Solute carrier family 6 member 13)
206	*SPATA2L*	Q8IUW3	Spermatogenesis-associated protein 2-like protein (SPATA2-like protein)
207	*CYP27A1*	Q02318	Sterol 26-hydroxylase, mitochondrial (EC 1.14.15.15) (5-beta-cholestane-3-alpha,7-alpha,12-alpha-triol 26-hydroxylase) (Cytochrome P-450C27/25) (Cytochrome P450 27) (Sterol 27-hydroxylase) (Vitamin D(3) 25-hydroxylase)
208	*SDHA*	P31040	Succinate dehydrogenase [ubiquinone] flavoprotein subunit, mitochondrial (EC 1.3.5.1) (Flavoprotein subunit of complex II) (Fp)
209	*SDHB*	P21912	Succinate dehydrogenase [ubiquinone] iron-sulfur subunit, mitochondrial (EC 1.3.5.1) (Iron-sulfur subunit of complex II) (Ip)
210	*SUCLG1*	P53597	Succinate–CoA ligase [ADP/GDP-forming] subunit alpha, mitochondrial (EC 6.2.1.4) (EC 6.2.1.5) (Succinyl-CoA synthetase subunit alpha) (SCS-alpha)
211	*SUCLG2*	Q96I99	Succinate–CoA ligase [GDP-forming] subunit beta, mitochondrial (EC 6.2.1.4) (GTP-specific succinyl-CoA synthetase subunit beta) (G-SCS) (GTPSCS) (Succinyl-CoA synthetase beta-G chain) (SCS-betaG)
212	*SDC3*	O75056	Syndecan-3 (SYND3)
213	*TTC27*	Q6P3X3	Tetratricopeptide repeat protein 27 (TPR repeat protein 27)
214	*TTC28*	Q96AY4	Tetratricopeptide repeat protein 28 (TPR repeat protein 28) (TPR repeat-containing big gene cloned at Keio)
215	*TTC9B*	Q8N6N2	Tetratricopeptide repeat protein 9B (TPR repeat protein 9B)
216	*TRAF6*	Q9Y4K3	TNF receptor-associated factor 6 (EC 2.3.2.27) (E3 ubiquitin-protein ligase TRAF6) (Interleukin-1 signal transducer) (RING finger protein 85) (RING-type E3 ubiquitin transferase TRAF6)
217	*TLR2*	O60603	Toll-like receptor 2 (EC 3.2.2.6) (Toll/interleukin-1 receptor-like protein 4) (CD antigen CD282)
218	*TRABD*	Q9H4I3	TraB domain-containing protein (Protein TTG2)
219	*ENY2*	Q9NPA8	Transcription and mRNA export factor ENY2 (Enhancer of yellow 2 transcription factor homolog)
220	*TFAP4*	Q01664	Transcription factor AP-4 (Activating enhancer-binding protein 4) (Class C basic helix-loop-helix protein 41) (bHLHc41)
221	*GUF1*	Q8N442	Translation factor GUF1, mitochondrial (EC 3.6.5.-) (Elongation factor 4 homolog) (EF-4) (GTPase GUF1) (Ribosomal back-translocase)
222	*MTIF3*	Q9H2K0	Translation initiation factor IF-3, mitochondrial (IF-3(Mt)) (IF-3Mt) (IF3(mt)) (IF3mt)
223	*TMEM141*	Q96I45	Transmembrane protein 141
224	*TMEM70*	Q9BUB7	Transmembrane protein 70, mitochondrial
225	*TMPPE*	Q6ZT21	Transmembrane protein with metallophosphoesterase domain (EC 3.1.-.-)
226	*TNRC18*	O15417	Trinucleotide repeat-containing gene 18 protein (Long CAG trinucleotide repeat-containing gene 79 protein)
227	*TRMT10C*	Q7L0Y3	tRNA methyltransferase 10 homolog C (HBV pre-S2 trans-regulated protein 2) (Mitochondrial ribonuclease P protein 1) (Mitochondrial RNase P protein 1) (RNA (guanine-9-)-methyltransferase domain-containing protein 1) (Renal carcinoma antigen NY-REN-49) (mRNA methyladenosine-N(1)-methyltransferase) (EC 2.1.1.-) (tRNA (adenine(9)-N(1))-methyltransferase) (EC 2.1.1.218) (tRNA (guanine(9)-N(1))-methyltransferase) (EC 2.1.1.221)
228	*PUS1*	Q9Y606	tRNA pseudouridine synthase A (EC 5.4.99.12) (tRNA pseudouridine(38–40) synthase) (tRNA pseudouridylate synthase I) (tRNA-uridine isomerase I)
229	*TNFRSF19*	Q9NS68	Tumor necrosis factor receptor superfamily member 19 (TRADE) (Toxicity and JNK inducer)
230	*YARS2*	Q9Y2Z4	Tyrosine–tRNA ligase, mitochondrial (EC 6.1.1.1) (Tyrosyl-tRNA synthetase) (TyrRS)
231	*OTULIN*	Q96BN8	Ubiquitin thioesterase otulin (EC 3.4.19.12) (Deubiquitinating enzyme otulin) (OTU domain-containing deubiquitinase with linear linkage specificity) (Ubiquitin thioesterase Gumby)
232	*UBE3C*	Q15386	Ubiquitin-protein ligase E3C (EC 2.3.2.26) (HECT-type ubiquitin transferase E3C) (HectH2)
233	*CMPK2*	Q5EBM0	UMP-CMP kinase 2, mitochondrial (EC 2.7.4.14) (Nucleoside-diphosphate kinase) (EC 2.7.4.6)
234	*C7orf31*	Q8N865	Uncharacterized protein C7orf31
235	*KIAA0930*	Q6ICG6	Uncharacterized protein KIAA0930
236	*VASN*	Q6EMK4	Vasorin (Protein slit-like 2)
237	*ACADVL*	P49748	Very long-chain specific acyl-CoA dehydrogenase, mitochondrial (VLCAD) (EC 1.3.8.9)
238	*VWA5B1*	Q5TIE3	von Willebrand factor A domain-containing protein 5B1
239	*XPNPEP3*	Q9NQH7	Xaa-Pro aminopeptidase 3 (X-Pro aminopeptidase 3) (EC 3.4.11.9) (Aminopeptidase P3) (APP3)
240	*ZCCHC24*	Q8N2G6	Zinc finger CCHC domain-containing protein 24
241	*ZNF428*	Q96B54	Zinc finger protein 428 (Enzyme-like protein PIT13)
242	*ZNF625*	Q96I27	Zinc finger protein 625
243	*ZNF782*	Q6ZMW2	Zinc finger protein 782
244	*HKR1*	P10072	Zinc finger protein 875 (Krueppel-related zinc finger protein 1) (Protein HKR1)

Remarkably, both EVs and mitochondria are acknowledged to be the crucial players in kidney stone formation [25, 130, 131, 148, 159–161]. As such, using anti-oxidants and/or other means of preservation of mitochondrial functions are expected to be one of the ideal strategies for KSD prevention [129, 162–166]. Although mitochondrial dynamics and mitophagy have been investigated and proposed as the main processes in the MQC system in many diseases [167–170], their roles in KSD remain underinvestigated [171]. Interestingly, MDVs have been demonstrated as the novel key player in the MQC system that is the main mechanism for mitochondrial homeostasis and mitochondrial stress response in several diseases, including kidney disorders [5, 171]. Recent studies of MDVs have demonstrated that MDVs can reduce inflammatory response and preserve healthy mitochondria in mild stress, leading to reduction of tissue injury [169–172].

The beneficial roles of MDVs are mediated via the MQC system to place a limit on mitochondrial dysfunction under the normal and mild stress conditions [8, 10, 40]. Also, they are the substitutable machineries to replace the other impaired processes in the MQC system such as mitochondrial dynamics and mitophagy [1, 8, 10, 40, 46]. Thus, the damaged mitochondrial components induced by oxidative stress, including oxidized mtDNA, proteins and lipids, are eradicated from the unhealthy mitochondria by MDVs to restore the healthy mitochondria inside the cells [3, 7, 22, 100, 173]. These processes can further reduce oxidative stress and prevent cell death. Besides, MDVs can remove the excessive mtROS and other proinflammatory molecules that tend to trigger proinflammatory signaling and cytokine production [21, 39, 100]. Therefore, MDVs formation is considered as the rapid and foremost protective response to prevent mitochondrial dysfunction, cell death and tissue inflammation/injury under the oxidative stress condition.

**Fig. 2:**
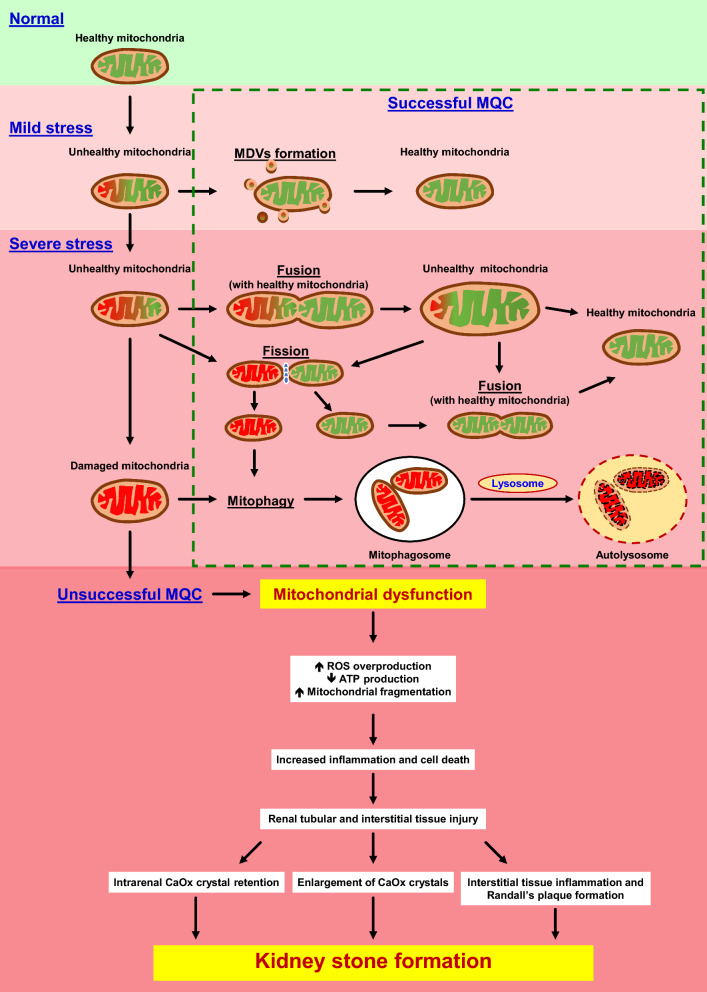
Roles of MDVs and MQC system in KSD. At early stage of oxidative stress with mild mitochondrial damage, MDVs (as a part of the MQC system) are formed to eliminate the malfunctioned mitochondrial components. Under severe oxidative stress condition, mitochondrial dynamics (fission/fusion) and mitophagy are predominantly activated to rescue mitochondrial structure and functions. When the MQC system is overwhelmed by extremely severe oxidative stress, mitochondrial dysfunction occurs, leading to ROS overproduction, mitochondrial degradation, inflammation, cell death, and renal tubulointerstitial injury. All these detrimental derangements lead to CaOx crystal deposition, growth, aggregation, nidus formation, Randall’s plaque development and, finally, kidney stone formation

MDVs carry not only the damaged mitochondrial components but also the healthy mitochondrial compartments that can be transferred and released to the unhealthy mitochondria for maintaining cellular functions and survival. Previous studies have demonstrated that MDVs can transport functional mtDNA, mitochondrial matrix, IMM, OMM and fragmented mitochondria to other malfunctioned mitochondria inside the same cell or outside (adjacent cells) [41, 174–179]. Recently, the in vitro synthesis of MDVs has been developed and applied for reduction of cell apoptosis [2, 40, 73]. In the study of myocardial ischemic/hypoxic injury, administration of exogenous (synthetic) MDVs has been demonstrated to serve as the new and effective therapeutic strategy [39, 57]. Most of previous studies have suggested that both intracellular and extracellular MDVs have the protective roles against mitochondrial damage, oxidative stress and tissue/organ injury. Although the clear evidence for the beneficial roles of MDVs in KSD prevention is not currently available, we propose that MDVs would also play such protective role to cope with mitochondrial dysfunction and oxidative stress that are common in KSD (Fig. 2). Therefore, MDVs may serve as the novel therapeutic target to prevent KSD related to mitochondrial dysfunction and oxidative stress as described above.

Recently, uEVs serve as the important source for biomarker discovery in several kidney and non-kidney diseases [180–182]. In KSD, a recent proteome study of urinary exosomes has demonstrated greater levels of proteins in S100A family (S100A8, S100A9 and S100A12) in urinary exosomes derived from stone patients compared with those from healthy individuals [183]. Therefore, these exosomal S100A proteins may serve as the biomarkers for KSD. As MDVs share similar proteome and lipidome profiles with EVs, another important role of MDVs in diagnostics and prognostics of KSD should be more extensively investigated.

## Conclusions and perspectives

MDVs are one of the most significant players in the MQC system to preserve mitochondrial structure and functions in normal and mild oxidative stress conditions [1, 4, 5, 10]. MDVs are also involved in various diseases, particularly cardiovascular diseases [20, 21] and neurodegenerative disorders [22–24]. In the kidney, the abundance of mitochondria per cell and their functions are critical for maintaining renal tubular cell functions along the nephron. During oxidative stress, mitochondrial dysfunction and tubulointerstitial inflammation occur and induce kidney stone formation [25, 116, 184]. Therefore, mitochondria are the key player in KSD development. The MQC system serves as the central machinery for mitochondrial homeostasis to prevent cell death and tissue injury [5, 6, 172]. MDVs, as the essential compartment of the MQC system [1–4], play the protective roles to rescue the malfunctioned mitochondria during mild stress to preserve their normal structure and functions (Fig. 2).

At early stage of oxidative stress with mild mitochondrial damage, MDVs formation is the rapid and effective process for preserving mitochondrial functions. Under severe oxidative stress condition, mitochondrial dynamics (fission/fusion) and mitophagy are predominantly activated to rescue mitochondrial structure and functions [185–188]. Additionally, MDVs generation can be also triggered as the major MQC machinery to cope with unhealthy mitochondria when mitophagy is unsuccessful for eliminating the damaged mitochondria or mitochondrial fission/fusion fails to recover the mitochondrial structure and functions [1, 8, 10, 40]. When the MQC system is overwhelmed by extremely severe oxidative stress, mitochondrial dysfunction occurs, leading to ROS overproduction, mitochondrial degradation, inflammation, cell death, and renal tubulointerstitial injury. All these detrimental derangements lead to CaOx crystal deposition, growth, aggregation, nidus formation, Randall’s plaque development and, finally, kidney stone formation [25, 129, 146, 147] (Fig. 2).

Nevertheless, the current knowledge on roles of MDVs under physiologic and pathophysiologic conditions remains incomplete. Several advanced methods/techniques have been continuously developed to further clarify the MDVs biology and functions, such as MDVs formation mechanisms, subtypes, specific contents, targets, and diagnostic/therapeutic potential [68, 75, 78, 189]. As MDVs seem to be more dynamic than we initially anticipated, isolation and purification of MDVs also need further development to obtain the specific subtype(s) of the purified MDVs. Differential ultracentrifugation is the primary method for MDVs isolation but still requires further improvement for better yield and higher purity [68, 75]. After isolation, characterizations can be done by morphological examination using high-resolution electron microscopy [19, 68]. To validate MDVs subtypes, proteome and lipidome studies should be performed followed by immunodetection [19, 68].

Recent evidence has demonstrated the therapeutic potential of MDVs in several diseases, including Parkinson’s disease [190], Down syndrome [68], Alzheimer’s disease [191], and myocardial ischemia [39, 192]. Interestingly, the synthetic MDVs have been successfully generated in vitro [39, 193–196]. These synthetic (exogenous) MDVs can be produced by activating the isolated mitochondria by chemical reaction, energy regenerating system, or mild stress-inducing reagents [39, 193]. This technique is therefore promising for further characterizations of MDVs and for developing MDVs-based therapeutic strategies in various diseases, including KSD.

In addition to the therapeutic/preventive potential, MDVs also have a promising role in diagnostics/prognostics of KSD. Future studies on biomarker discovery for KSD should focus on MDVs and their specific types. For example, at an initial phase of kidney stone development with mild stress condition or slight tissue injury, mtDNA and mtROS can be excreted through PDH^+^-MDVs and transferred to blood circulation and/or urine. Therefore, identification of urinary PDH^+^-MDVs containing mtDNA or mitochondrial proteins, together with evidence of supersaturation of crystalline compounds in the urine would yield an early biomarker for KSD.

## Data Availability

All data generated or analyzed during this study are included in this published article and are also available from the corresponding author on reasonable request.
