# Correction: Mitochondria-derived vesicles and their potential roles in kidney stone disease

**DOI:** 10.1186/s12967-023-04299-w

**Published:** 2023-07-11

**Authors:** Sakdithep Chaiyarit, Visith Thongboonkerd

**Affiliations:** Medical Proteomics Unit, Research Department, Faculty of Medicine Siriraj Hospital, Mahidol University, 6th Floor, SiMR Building, 2 Wanglang Road, Bangkoknoi, Bangkok, 10700 Thailand

**Correction: Journal of Translational Medicine (2023) 21:294** 10.1186/s12967-023-04133-3

Following publication of the original article [1], we have been notified that the Funding note was mentioned incorrectly. It should be as follows:

This study was supported by National Research Council of Thailand (NRCT) and Mahidol University (Grant No. N42A650369).

The original article [1] has been updated.
